# Digital Technologies and Data Science as Health Enablers: An Outline of Appealing Promises and Compelling Ethical, Legal, and Social Challenges

**DOI:** 10.3389/fmed.2021.647897

**Published:** 2021-07-08

**Authors:** João V. Cordeiro

**Affiliations:** ^1^Public Health Research Centre, NOVA National School of Public Health, Universidade NOVA de Lisboa, Lisboa, Portugal; ^2^Comprehensive Health Research Center, Universidade NOVA de Lisboa, Lisboa, Portugal; ^3^Centro Interdisciplinar de Ciências Sociais, Lisboa, Portugal

**Keywords:** digital health, ethics, law, artificial intelligence, telemedicine, big data, patient–doctor relationship

## Abstract

Digital technologies and data science have laid down the promise to revolutionize healthcare by transforming the way health and disease are analyzed and managed in the future. Digital health applications in healthcare include telemedicine, electronic health records, wearable, implantable, injectable and ingestible digital medical devices, health mobile apps as well as the application of artificial intelligence and machine learning algorithms to medical and public health prognosis and decision-making. As is often the case with technological advancement, progress in digital health raises compelling ethical, legal, and social implications (ELSI). This article aims to succinctly map relevant ELSI of the digital health field. The issues of patient autonomy; assessment, value attribution, and validation of health innovation; equity and trustworthiness in healthcare; professional roles and skills and data protection and security are highlighted against the backdrop of the risks of dehumanization of care, the limitations of machine learning-based decision-making and, ultimately, the future contours of human interaction in medicine and public health. The running theme to this article is the underlying tension between the promises of digital health and its many challenges, which is heightened by the contrasting pace of scientific progress and the timed responses provided by law and ethics. Digital applications can prove to be valuable allies for human skills in medicine and public health. Similarly, ethics and the law can be interpreted and perceived as more than obstacles, but also promoters of fairness, inclusiveness, creativity and innovation in health.

## Introduction

Innovative solutions to both classic and emergent medical problems have resulted from the impact of the digital revolution on healthcare (1, 2). Prominent examples include telemedicine, electronic health records, wearable, implantable, injectable and ingestible medical devices, health mobile apps, and the application of artificial intelligence (AI) algorithms to health settings (3). Correspondingly, computer power, interconnectivity and storage capacity, have potentiated the collection, analysis, and sharing of health data. These advancements, coupled with the expansion of data generation capabilities have led to an era of big data in healthcare, which promises to facilitate timely and precise healthcare interventions (4, 5). In order to achieve this aim, the extraction of knowledge from big data through the interdisciplinary work of data science is fundamental (6).

Better healthcare quality as a result of digital health applications and data science methods is an appealing promise, which also elicits significant ethical, legal, and social challenges (Table 1). This article aims to outline this dichotomy, with a limited focus on the examples of telemedicine and AI, which are two specific and interconnected areas in expansion.

**Table 1:** Summary of relevant ethical, legal, and social issues (ELSI) raised by digital technologies and health data processing in healthcare.

**Digital health ELSI**
**Ethical**
• Promotion of patient autonomy and empowerment
• Design, obtainment, and interpretation informed consent
• Identity confirmation and authentication
• Achieving fair distribution of risks, benefits, and costs
• Guaranteeing quality of care
• Strengthening the doctor–patient relationship
• Assuring continuity of care
• Defining professional duties and responsibilities
• Maintaining confidentiality
• Patient-generated health data
• Direct to consumer telemedicine and unsolicited requests for diagnosis and individual health management
• Dehumanization of care
• Moral status and ethical judgement of machines
• Human nature, *quantified-self* and *technological singularity*
**Legal**
• Appropriateness, coherence, and accessibility of regulation, including inconsistencies in interpretation by oversight bodies
• Assessment of validity, utility and quality of products, services, strategies, and interventions
• Data protection rights (including privacy by default, privacy by design, data destruction policies, the right to know and the right not to know)
• Data access, return of information and non-discrimination
• Data ownership rights, fair, transparent, and harmonized data sharing rules
• Compliance standards, oversight, and sanctions
• Broader data and device security issues
• Jurisdiction and licensure for telemedicine
**Social**
• Level of public participation and awareness
• Digital literacy levels of patients and health professionals
• Academic curricula adequacy (upgrades and updates)
• Limits to privacy and confidentiality in health
• Inequality and social stigma
• Lifestyle changes and adoption of healthy behaviors
• Impact on health access (economic, geographical, and informational)

*Despite categorization, issues are mainly hybrid in nature*.

In broad terms, telemedicine and telehealth consist in the practice of healthcare through information and telecommunication systems (7). This branch of digital health has had notorious growth in the last years and particularly during the COVID-19 pandemic (8–10). Its applications include, but are not restricted to, real-time health consultations from a distance, remote health data collection, analysis, interpretation, and monitoring, and digital interactions with health assistants, including virtual ones (7). Accordingly, these subjects have deserved particular ethical, legal and scholar attention in recent years (7, 11–13).

In parallel, AI applications in healthcare have gathered significant interest (14, 15). These include, but are also not restricted to, analysis of health data to predict health events and outcomes, check symptoms and improve diagnosis, suggest preventive strategies, design and develop new medicines, improve the organization and conduction of clinical trials, enhance patient experiences, and advance the structure and intelligibility of electronic health records (5, 14, 16–19). Consequently, the influence of AI and machine learning in the health sector is projected to expand and affect the work of healthcare professionals, the efficiency of health systems, and the capacity of patients to interpret their own health data (18, 20, 21). Similarly, awareness about the ethical, legal and social dimensions of AI broadened (22–25), which will hopefully translate into better regulation (26–28).

With a balanced view between promises and challenges and telemedicine and AI as examples, this article aims to provide a succinct review of the main ethical, legal and social implications (ELSI) of the adoption of digital technologies and the processing of health data in medicine and public health.

## Methods

The main topics highlighted in this article resulted from an initial literature search using electronic platforms *PubMed* and *Google Scholar* and general search terms (corresponding to the article keywords in combination with “ethics,” “law,” and “ELSI”). Additional references were identified by backward and forward citation chaining. Finally, articles from scholars known to the author were also analyzed to complement the analysis.

## ELSI of the Adoption of Digital Technologies in Healthcare

### Trust, Quality, and the Doctor–Patient Relationship

Trust is a fundamental and reciprocal value in healthcare. To obtain guidance and care, patients trust health professionals in a context of asymmetrical information (29). Conversely, health professionals trust patients to describe their individual experience and their medical history, as well as adhering to recommended behaviors and treatments (30). Therefore, the doctor–patient relationship is based on mutual trust, which is fundamental to ensure quality of care (31, 32). Admittedly, the uptake of digital technologies in healthcare can affect the doctor–patient relationship by reducing human contact and proximity (33). This effect has been substantially debated in the context of telemedicine (9, 12, 34, 35). In particular, the possible devaluation of the importance of continuous face-to-face interactions between patients and doctors, of non-verbal cues and of established ways of building empathy and rapport for economical or efficiency reasons have been highlighted (9). Notably, the impact of digital technologies (and telemedicine in particular) on the doctor-relationship might be very different depending on medical specialty. For example, it might be low for some interactions in dermatology, and completely reshape relations in the context of mental health specialties (35). Likewise, specific functions (image analysis/evaluation of health parameters vs. communication of diagnosis, for example) might also be impacted differently. These considerations reinforce the need to value patient context and preferences in order to improve quality in health, as superficial relationships (even if quantitatively informed) might lead to superficial care.

In a context of increased reliance on digital technologies, the trustworthiness of digital services and goods is fundamental to preserve the value of trust, to strengthen the doctor–patient relationship and increase healthcare quality (36). To achieve these goals, it is essential to rigorously assess the analytical validity and clinical validity and utility of digital technologies (37, 38). Particularly, validity assessment must consider scientific standards for market clearance and authorization, licensing and periodical evaluation, definition and enforceability of quality control norms, and professional requirements for usage and operation (including training, registration and authentication rules). However, despite ongoing efforts, audit and certification procedures are often variable, opaque and incompatible with the rhythm of technological progress (37, 39–41). Furthermore, clinical utility must be critically estimated and, subsequently, communicated to users, which involves the capacity to properly perceive and transmit technology's benefits and risks, as well as uncertain notions such as probability and variance. These issues, together with legal liability clarification and definition of malpractice norms across different geographies and jurisdictions, have been clearly identified as fundamental to guarantee that quality of care and patient safety are protected and hopefully improved during a sharp uptake of telemedicine and telehealth (8, 9, 34, 35).

Different health stakeholders will deliberate and define relations of trust differently (42). Nonetheless, in broad terms, the recommendation or adoption of subpar digital health services and products risks fostering mistrust in healthcare professionals, institutions and systems, which might ultimately affect the whole digital health field and the broader scientific endeavor (43, 44). As an example, paradigm cases like *Theranos*, in which a hyped promise to revolutionize the blood tests industry turned out to be fraudulent, illustrate the damage that can result from the lack of adequate scrutiny (45). Digital health has lessons to learn from this and other similar cases. Ultimately, the implementation of rigorous, updated and intelligible assessment models is a key component of the digital health promise to promote and advance ethics, evidence, and value-based healthcare (46).

In particular, the introduction of AI and machine learning algorithms in healthcare provides a relevant illustration of the growing need for dedicated assessment and validation. Although progress in the area of deep neural networks allows for assessment of algorithmic capacity using synthetic data, including medical images (47), peer-reviewed real-world clinical data must not be abandoned if poor accuracy or undetermined clinical utility are to be avoided (18). Cases like the IBM Watson-mediated promotion of unsafe and incorrect treatment recommendations to hospitals and medical doctors globally, illustrate how risky it is to adopt AI-based approaches without proper validation, particularly as these flaws can potentially affect large numbers of patients in a short timespan, severely affecting the elements of trust and quality in healthcare (48).

Finally, the extreme case of superficiality in contemporary and future medicine, some argue, is machinal healthcare (33). In fact, it might be precisely in healthcare that dehumanization might prove more costly as human vulnerability, hope, suffering, dependence and, ultimately, death are at stake (49). In accordance, healthcare practice extends beyond technical analysis to include ethics and morality. Interestingly, the competence of machines for moral reasoning, judgement and decision-making is a developing discussion (50–52). Either way, it would reinforce trust, promote quality and strengthen the doctor patient-relationship if digital health tools, and AI in particular, provide stronger incentives for healthcare professionals to focus on caring, compassion, and communication. These are fundamental skills, which are perceived by patients to be in decline (33, 53).

### Transparency, Bias, and Exclusion

In order to achieve its highest aims while preserving trust, the uptake of digital technologies in healthcare must be transparent (40). In a world where data can be artificially created, it is increasingly important that the non-human dimensions of healthcare are disclosed, including the usage of models and algorithms, both in and outside the context of telehealth (9, 14, 15, 54). Therefore, medical decisions supported by digital technologies should be more transparent and understandable, in order to simultaneously guarantee accountability and avoid patient disenfranchizement and exclusion. For example, the incapacity to understand and scrutinize algorithm decision-making leading to less transparency, a challenge known as the black box problem, is currently subject to intense debate including in the healthcare context (55, 56). Notably, the “right to explanation” of algorithm decisions and requirements for human intervention are legally established in different jurisdictions (57). In contrast, some authors view a degree of uncertainty as inevitable and perceive these conditions as obstacles to progress (18, 58). Nonetheless, common ground can be found in the need for better integration of scientific disciplines to surpass hyper technical discussions (and limited understanding and explainability) in contemporary medicine in the context of digital health in general, and AI in particular. Consequently, adaptation of academic curricula to digital health developments should be prioritized (59). In parallel, the emergence of new health skills or professions that oversee the development of common languages, intersect different disciplines, assist in science implementation and facilitate interactions between different stakeholders is likely (60).

On a separate yet related note, flawed algorithms can feed into human bias and potentiate discrimination (61, 62). Moreover, as the widespread use of facial recognition technology (FRT) edges closer, questionable studies portraying facial traits as proxies for different characteristics (including economic condition, emotional status, and sexual orientation) multiply, raising justified concerns about bias (63–65). The same is true for extrapolating conclusions from online digital behavior (66, 67). Healthcare is not foreign to this debate as FRT can be used to diagnose medical and genetic conditions, for example (68). Notoriously, the issue of AI bias is still open and evolving (69, 70). Nonetheless, it is unreasonable to expect that simply dehumanizing the flawed dimensions of healthcare will *per se* facilitate fairer health outcomes. Expectedly, feeding AI with biased data will lead to biased and unjust decisions (71, 72). Hence, AI implementation in healthcare demands great responsibility (73, 74). Additionally, data is context-dependent and biased context can result in biased conclusions, as studies have shown (75). Conversely, decontextualization of data can also result in algorithm bias, flawed decisions, and discrimination (75, 76). These are renewed arguments to keep fairness and justice at the center of the healthcare debate.

## ELSI of Digital Health Data Processing

### Autonomy, Consent, and Patient Participation

Grounded on the principle of autonomy, informed consent is a cornerstone of medical ethics. Valid informed consent requires a clear and precise acknowledgement of the situation, freedom from coercion (physical or psychological), and competence for decision making (or representation, in the case of minors and incompetent adults) (77). Notably, guaranteeing informed consent for heath research or care purposes, faces singular challenges in the digital era, including identity confirmation, remote evaluation of voluntariness, assessment of understanding levels and competence determination (78, 79).

Defining the scope of consent for health data processing is especially difficult. On one hand, single purpose consent is problematic as secondary uses are often necessary for research and care purposes and re-consent is impracticable (80). On the other hand, in an increasingly fluid ecosystem with expanding interactions between different stakeholders and infrastructures (hospitals, clinics, biobanks, research institutes, biotechnology, and pharma) and possible cycling of health data between health research and healthcare contexts, significant challenges are posed to classical informed consent models. Consequently, in alternative to otherwise open consent options, dynamic consent models have been proposed and justify continuous efforts of implementation (81, 82). Additionally, as health data anonymity is a commodity in an interconnected digital context, legal compliance, management of expectations and risk assessment and communication add extra pressure and complexity to the informed consent process (83–85). Furthermore, some types of health data (for example genetic data), might be shared by more than one person, blurring the limits of individual consent and rights while urging extra care in defining norms and interpreting the law (86).

Along with these challenges to informed consent, the issues of patient autonomy, participation and the doctor–patient relationship converge on other equally challenging digital health data trends. For example, health data can now be generated by patients themselves (*via* apps, wearables, and other digital means) (87). In parallel, healthcare interactions can be patient-initiated (requests of diagnosis and treatment, for example) (88). Thirdly, direct-to-consumer health services, including telemedicine and AI, are expanding (17, 89). These trends highlight the need to extract meaning and knowledge from large quantities of data, while protecting patients from misinformation, misjudgement and disenfranchizement (90). For example, individual and collective risks such as unjustified anxiety, false reassurance and overconsumption of scarce health resources are magnified by digital health illiteracy, neglect, or abandonment to excessive technicality (15, 17, 87, 91). Equally, confirming the accuracy of patient-generated health data and streamlining its integration with electronic health records should be harmonized if healthcare quality is to be promoted (92).

It is well-established that health illiteracy and the digital divide affect patient participation, possibly compromising access to healthcare (93–96). Therefore, to respect autonomy and promote patient participation, the most vulnerable (due to isolation, disability, age, illiteracy, and other factors) deserve special attention and protection. This implies rejecting a *one-size-fits-all* approach and tailoring digital healthcare encounters to individual needs and histories, which are told by different types of data. Among different digital health services and products, which can promote health data sharing and healthcare access, telemedicine has understandably gathered special recognition due to its proven capacity to extend healthcare access to isolated communities (35, 97). Nonetheless, challenges remain as some studies have also indicated that telemedicine services might contribute to medicalize the home context and end up worsening isolation and dependence in some cases (35). This fact further underlines the relevance of context-dependent assessment models and implementation efforts. Furthermore, as telehealth services grew quickly to meet demand in a context of reduced physical interactions such as the COVID-19 pandemic, one must not use the lack of immediate alternatives as an excuse for neglecting fundamental aspects of healthcare ethics. Particularly, the delimitation of professional responsibilities (clinical, administrative, and other) related with health data accessibility and sharing [including the clarification of End User License Agreements (EULAs)] and improved risk communication and cultural respect in digital interactions must be focused on in order to attribute real meaning to health data and achieve the highest hopes for this technology (9).

Finally, it is the human pondering of different alternatives that enriches the consent process and furthers patient participation and autonomy in healthcare. As machine learning algorithms gather more influence and prove to be increasingly autonomous, new challenges are posed to these ethical principles in the context of health data processing (98). Therefore, progress in healthcare should not consist in the promotion of machine autonomy at the expense of human autonomy. On the contrary, well-established human values in healthcare, such as integrity, conscientiousness and compassion must guide health data processing in a digital context and work as allies of digital health innovation.

### Privacy, Confidentiality, and Security

The issues of privacy, confidentiality and data protection are recognized as fundamental rights in most jurisdictions and are especially challenging for digital health (99–103). The old debate surrounding the erosion of privacy and confidentiality in health settings endures (104, 105). For example, the protection of electronic health records has been widely recognized as insufficient (106, 107). Furthermore, privacy protection, data access, interoperability, and quality of recorded data are recurrently reported as ELSI of digital health, including of telemedicine (9, 12, 34, 35). Undoubtedly, health data processing is essential for medical and scientific progress and should follow transparent, balanced and fair rules (108–112). In order to strengthen fundamental rights in the digital age and regulate the free movement of personal data, including health data, the EU has adopted the General Data Protection Regulation (GDPR) (113). This broad ranging legal document reinforced mechanisms of data protection, including more transparency and accountability, mandatory impact assessments, pan-European validation of codes of conduct, certification procedures, and more severe sanctions (114). Furthermore, rights of data subjects were enhanced, including a right of access and rights to information, explanation, rectification, erasure, restriction of processing, data portability, object and not to be subject to automated individual decision-making (113). Due to its broad scope and recent nature, it is still early to judge the impact of GDPR in the health area. Additionally, harmonization of health data flows across jurisdictions is still problematic. For example, the successive European Court of Justice *Schrems* cases (115), leading to successive annulments of legal agreements regulating EU-US data flows, have impacted health research and healthcare (116).

In parallel, health data security has become a serious concern as poor protection measures combined with high transactional value, exacerbate the risk of violations and damages (117, 118). Particular cybersecurity concerns emerge from the expansion of digital health, including telemedicine and the multiplication of interconnected sensors and medical devices (119), which has rightly deserved regulatory attention (120–123). Ultimately, healthcare institutions should see their data security infrastructure strengthened and recent technological progress can provide the tools for risk mitigation (124).

In complement to data protection measures, responsible use of data is key. Especially, projects with public notoriety demand greater responsibility if public trust is to be preserved. For example, cases such as the Google Deepmind collaboration with the UK NHS (125, 126), NHS England's care.data programme (127), or “Project Nightingale” in the US, in which patient data was accessed by commercial companies without informed consent, emphasize the need for transparency, communication and responsibility in order to guarantee the positive impact of data sharing on healthcare. In contrast, opacity extends power imbalances and unprotects citizens (128). Therefore, clear and fair health data ownership rules, beyond the traditional property approaches, should continue to be developed and harmonized. These should guarantee patient access to their data (129) while limiting access and usage by third parties without a compelling interest. Moreover, different strategies can be adopted to encourage responsible use of health data. For example, investing in healthcare ethics literacy programs, implementing validated codes of conduct (institutional, national, and international), refining deontology rules (for health professionals and data scientists), protecting whistleblowers of data mistreatment practices, setting fair procedures, and imposing dissuasive sanctions (disciplinary, legal, and social) for confirmed misconduct. Obviously, such strategies must include electronic health records and health data shared in telemedicine settings, processed by mobile apps and medical devices, as well as AI algorithms (130, 131).

## Discussion

The digital revolution has impacted different areas of society, including healthcare (19). At the core of this transformation is an increased capacity to process large quantities of health data using digital means (15, 16). Big data in healthcare originates from different sources, including biological and social determinants, health records, environmental signals, habits, and behaviors (19, 132, 133). Against this backdrop, telemedicine and telehealth services expanded significantly in recent years, particularly during the COVID-19 pandemic (10). Furthermore, AI applications are gaining ground in complementing even the most knowledgeable or skilled professionals (134, 135). Concomitantly, a new era of precision healthcare is promised, where the right individual and public intervention is available for the right patient or population at the right time (136–141).

In this context, the optimization of health data processing using digital means and the general uptake of digital technologies can rightly be perceived as health enablers. In parallel, compelling related ELSI must be considered and dealt with.

Expectedly, healthier activities and wiser health choices should result from better data science. In this sense, digitally-mediated health data processing can promote individual autonomy and patient empowerment (142–144). However, the adoption of healthy behaviors does not emerge linearly from better health information (which must be extracted from health data) as human decision making is complex and affected by context and cognitive biases, combining emotion and reason. Therefore, data-assisted decision making in healthcare, justifies a closer collaboration between healthcare professionals, decision, and data scientists and ethicists (15, 33). In fact, the links between health data and statistics literacy and healthcare quality is a classical debate, which is expected to intensify (145). Indeed, the adoption of digital technologies has the potential to improve the volume and quality of health data processing in order to expand knowledge to professionals and patients alike. However, a lack of common platforms and cross-disciplinary languages to deal with increasing technical complexity are significant challenges (146). Also, can technology, data and analytical models alone capture human vulnerability, suffering, fears, hopes and potential? Evidently not. Nonetheless, these can elevate the standard of care by providing healthcare professionals with invaluable (an otherwise inaccessible) information and knowledge, while alleviating the burden of repetitive and laborious tasks to focus on compassion and emotional connections, which are associated with the highest quality (15, 33). To this end, patient stories, particularly those of the most vulnerable, must be heard and understood and one must be mindful that health data misuse can contribute to misinformation, poorer care, reinforced exclusion or stigmatization. Notably, such risks are exacerbated if human health and disease are looked at from a purely quantitative lens (147–150). There is, however, cause for optimism as digital technologies can also be used to promote scientific robustness and tackle the very risks it potentially generates (151–154).

Balanced health data processing and usage of digital technologies to improve healthcare quality is a matter of public interest. Presently, there is significant data access asymmetries between citizens, corporations and governments (128). Therefore, urgent efforts are necessary to reach an inclusive and democratic deliberation leading to the simultaneous advancement of science and human rights. Importantly, digital technologies can advance the fulfillment of the human right to health. Specifically, they can improve the availability of health facilities, services and goods; increase the acceptability of practices (by incorporating medical ethics and approximating cultures); raise the quality of scientific and medical services, goods and professional skills; and promote access without discrimination (155, 156). The specific issue of fairness regarding access to digital technologies has been a key topic in the context of the accelerated uptake of telemedicine. This is particularly relevant as those who are more likely to benefit from this technology and its applications (isolated communities, including in rural areas) are also those who, predictably, are less capable of affording or using them (35). Therefore, public and individual interests must be properly balanced in order to maximize the potential of this technology while respecting human rights. Additionally, big data, telehealth, machine learning algorithms and the era of individual profiling might be ingredients for deeper discrimination and stigmatization (142). In summary, the positive application of digital technologies and data science in medicine and public health should promote, not defer progress in social justice.

In conclusion, the ELSI of the digital health field (Table 1) are compelling and proportional to the positive impact of digital technologies in healthcare. Consequently, normative orders such as law and ethics should act as beneficial limit-setters and promoters of just, creative and innovative realities. Accordingly, digital health ELSI convoke ethicists, legal scholars, patients, scientists, health professionals, health providers and payers, regulators, managers, and other decision makers to play a role in this fascinating field, which promises to decisively shape the way health and disease are perceived, assessed and managed in the future (157, 158).

## Author Contributions

The author confirms being the sole contributor of this work and has approved it for publication.

## Conflict of Interest

The author declares that the research was conducted in the absence of any commercial or financial relationships that could be construed as a potential conflict of interest.
